# Changes in gut microbiota and plasma metabolites in patients with symptomatic degenerative rotator cuff tendinopathy

**DOI:** 10.3389/fmicb.2026.1927912

**Published:** 2026-09-01

**Authors:** Yanzi Liu, Zheng Liu, Bin Zhang, Weijun Wang, Xuedong Tian, Wendan Cheng

**Affiliations:** 1 Department of Orthopaedics, The Second Affiliated Hospital of Anhui Medical University, Hefei, China; 2 Institute of Orthopaedics, Research Center for Translational Medicine, The Second Affiliated Hospital of Anhui Medical University, Hefei, China; 3 Department of Sports Medicine, Zibo Hospital of Traditional Chinese Medicine, Zibo, China

**Keywords:** 16S rRNA sequencing, degenerative rotator cuff tendinopathy, gut microbiota, gut-tendon axis, untargeted metabolomics

## Abstract

**Objective:**

To investigate alterations in gut microbiota and plasma metabolites in patients with symptomatic degenerative rotator cuff tendinopathy (dRCT), and to explore potential microbiota-mediated metabolic mechanisms involved in tendon degeneration.

**Methods:**

A case-control study with group-level matching for age, sex, and BMI was conducted, including 61 patients with symptomatic dRCT confirmed by clinical examination and magnetic resonance imaging and 61 healthy controls matched for age, sex, and body mass index. One RCT participant was subsequently excluded from all the final analyses due to insufficient sample volume. Fecal and plasma samples were collected for 16S rRNA amplicon sequencing and untargeted metabolomic analysis. PICRUSt2-based microbial functional prediction and microbiota–metabolite correlation analysis were performed to characterize gut microbial and metabolic alterations associated with dRCT.

**Results:**

Gut microbiota β-diversity differed significantly between dRCT patients and controls (*P* = 0.014). LEfSe analysis showed that the dRCT group was enriched in Bacteroidota and Pseudomonadota, as well as genera including Segatella, Megamonas, Klebsiella, Desulfovibrio, Agathobacter, and Alistipes. In contrast, controls were enriched in Bacillota and short-chain fatty acid-producing genera, including Gemmiger and the Eubacterium_ruminantium group. PICRUSt2 prediction indicated upregulation of pro-inflammatory microbial pathways in dRCT, including lipopolysaccharide biosynthesis, flagellar assembly, vitamin B6 metabolism, and riboflavin metabolism, whereas controls showed higher activity in amino acid synthesis and tissue repair-related pathways, including valine/leucine/isoleucine biosynthesis, lysine biosynthesis, and cysteine and methionine metabolism. Untargeted metabolomics identified 309 differential metabolites in positive ion mode and 226 in negative ion mode. KEGG enrichment revealed significant alterations in tryptophan metabolism and ferroptosis pathways in positive ion mode, and changes in unsaturated fatty acid biosynthesis, bile secretion, tryptophan metabolism, and steroid hormone biosynthesis in negative ion mode. Correlation analysis showed that Segatella and Megamonas were negatively correlated with indole-3-acetaldehyde and vitamin E, while Gemmiger was positively correlated with Quininic acid.

**Conclusion:**

To our knowledge, this is the first human multi-omics study systematically investigating the potential “gut–tendon axis”. Patients with dRCT exhibited gut microbiota dysbiosis characterized by enrichment of pro-inflammatory bacteria and a divergent shift among SCFA/butyrate-producing bacteria, with net depletion of the predominant taxa. These alterations may be associated with tendon degeneration through gut-derived endotoxin production, reduced tryptophan-to-indole conversion capacity, and ferroptosis-related oxidative stress.

## Introduction

Degenerative rotator cuff tendinopathy (dRCT) is one of the most common shoulder joint disorders encountered in clinical practice, characterized by shoulder pain, restricted range of motion, and decreased muscular strength, significantly affecting patients' quality of life and work capacity (Bedi et al., 2024; Wang et al., 2024). Epidemiological studies indicate that the prevalence of degenerative rotator cuff tendinopathy exceeds 15% in individuals over 60 years of age and increases significantly with advancing age (Minagawa et al., 2013). Despite continuous improvements in surgical techniques, the retear rate remains substantially high (20%−60%), suggesting that local mechanical repair cannot fully address the systemic pathogenic mechanisms underlying this condition (Baur et al., 2025; Longo et al., 2021). Emerging evidence demonstrates that the pathophysiology of degenerative rotator cuff tendinopathy is highly complex, involving the synergistic effects of multiple factors including mechanical wear, age-related degeneration, local ischemia, oxidative stress, and systemic chronic inflammation (Millar et al., 2021). However, a comprehensive and systematic understanding of its etiopathogenic mechanisms remains lacking, which constrains the development of effective prevention and intervention strategies.

In recent years, the regulatory role of the gut microbiota in musculoskeletal system disorders has gained substantial attention. Accumulating evidence confirms that gut dysbiosis is closely associated with osteoporosis, osteoarthritis, rheumatoid arthritis, and sarcopenia, with mechanisms involving immune modulation, inflammatory signaling, and short-chain fatty acid (SCFA)-mediated metabolic regulation (Li et al., 2021; Liu et al., 2023; Pang et al., 2025). The gut microbiota regulates the host's systemic inflammatory status, oxidative stress levels, and nutritional metabolism through the production of bioactive molecules such as butyrate and propionate (SCFAs), secondary bile acids, and tryptophan metabolites, thereby exerting profound effects on the homeostasis of distal tissues (Agus et al., 2021).

As a mechanically sensitive dense connective tissue, the tendon's extracellular matrix (ECM) homeostasis depends on a delicate balance between collagen synthesis and degradation. Systemic inflammatory mediators can activate NF-κB and MAPK signaling pathways in tendon cells, promoting excessive expression of matrix metalloproteinases (MMPs), accelerating ECM degradation, and inducing tendon degeneration (Millar et al., 2017). Lipopolysaccharide (LPS), the endotoxin from gram-negative intestinal bacteria, can translocate across the damaged intestinal barrier into systemic circulation and activate Toll-like receptor 4 (TLR4)-mediated inflammatory pathways, serving as a core driving factor of “gut-derived systemic inflammation” (Mohr et al., 2022; Tume et al., 2025). Furthermore, the effects of gut-derived metabolites such as tryptophan metabolites, SCFAs, and polyunsaturated fatty acids on tendon cell biology remain inadequately explored.

Currently, research examining the relationship between gut microbiota and tendon diseases is extremely limited, with available evidence primarily derived from animal models (Wang et al., 2025). To help fill this knowledge gap, we conducted this study. This investigation employs a case-control design with group-level matching, integrating 16S rRNA amplicon sequencing, untargeted metabolomic analysis, PICRUSt2 functional prediction, and microbiota-metabolite correlation analysis. Our aim is to systematically characterize the gut microbiota-metabolomic profile in patients with symptomatic degenerative rotator cuff tendinopathy, construct a tripartite network centered on “gut microbiota-metabolome-dRCT,” elucidate the alterations in gut microbiota and plasma metabolites in these patients, and provide new therapeutic targets for clinical treatment.

## Materials and methods

### Study subjects and design

A case-control study design with group-level matching for age, sex, and BMI was employed. The case group (RCT group, *n* = 61) consisted of patients with symptomatic degenerative rotator cuff tendinopathy confirmed by clinical examination and MRI. The control group (Control group, *n* = 61) comprised healthy volunteers recruited to approximate the RCT group's distribution of age, sex, and BMI. Participants were enrolled between October 2024 and February 2026; one additional RCT participant was excluded from all the final analyses due to insufficient sample volume for sequencing/metabolomic testing, yielding a final analytic sample of 121 participants. All participants provided written informed consent. The study protocol was approved by the Ethics Committee of Zibo Hospital of Traditional Chinese Medicine [Zibo Orthopedic Hospital; Approval number: (2024-070)]. Inclusion criteria: (1) Age ≥ 45 years; (2) Presenting with clinical symptoms of shoulder pain and functional limitation confirmed by positive clinical examination findings (positive Neer sign, Hawkins sign, Jobe test, etc.); (3) MRI findings demonstrating rotator cuff tendon degeneration, including tendinitis, abnormal intra-tendinous signal intensity, or partial tendon tear; (4) No history of shoulder trauma; (5) No use of antibiotics, probiotics, glucocorticoids or immunosuppressive agents within the preceding 3 months; (6) No active gastrointestinal infection. Exclusion criteria: (1) Active infectious disease; (2) Inflammatory bowel disease, irritable bowel syndrome, or other gastrointestinal disorders; (3) Autoimmune diseases (rheumatoid arthritis, systemic lupus erythematosus, etc.); (4) Malignancy; (5) History of shoulder surgery; (6) Pregnancy or lactation; (7) Severe cardiac, hepatic, or renal dysfunction; (8) Hypertension, diabetes mellitus, hyperlipidemia, or other metabolic disorders.

### Sample collection and processing

Fecal samples (approximately 5 g of fresh feces) were collected in sterile collection tubes following the first morning bowel movement after an overnight fast and immediately stored at −80 °C. Peripheral blood samples were collected from all participants in the fasting state in the early morning. After centrifugation at 3,000 rpm for 10 min, plasma was separated, aliquoted, and stored at −80 °C for subsequent metabolomic analysis.

### 16 S rRNA amplicon sequencing and analysis

Total genomic DNA was extracted from fecal samples using the Fecal Genomic DNA Extraction Kit (TIANGEN Biotech, Beijing, China; catalog no. DP328), following the manufacturer's protocol. The V3–V4 hypervariable regions of the 16S rRNA gene were amplified by PCR using the primer pair (341F 5′-CCTAYGGGRBGCASCAG3′) and (806R 5′-GGACTACNNGGGTATCTAAT3′), followed by paired-end sequencing on the Illumina NovaSeq platform (2 × 250 bp). Raw sequencing data underwent quality control filtering using fastp (v0.23.1), paired-end sequences were assembled using FLASH software (v1.2.11; minimum overlap 10 bp, maximum mismatch rate 0.2), and denoising was performed within the QIIME2 framework using DADA2, generating amplicon sequence variants (ASVs). Each ASV was taxonomically annotated using the classify-sklearn algorithm with the SILVA 138.2 reference database. Alpha diversity indices (Shannon, Simpson, etc.) were calculated within the QIIME2 framework; beta diversity was assessed using weighted UniFrac distance and visualized *via* principal coordinate analysis (PCoA); inter-group differences in community structure were tested using ANOSIM and PERMANOVA (Adonis). Differential taxonomic units were identified using LEfSe (LDA score threshold > 2). Functional pathway prediction was performed using PICRUSt2.

### Untargeted metabolomic analysis

*Sample pretreatment:* One hundred microliters of plasma sample were placed in an Eppendorf tube, to which 400 μL of 80% methanol aqueous solution was added. After vortexing, the sample was incubated on ice for 5 min, then centrifuged at 15,000 × g for 20 min at 4 °C, and the supernatant was collected. A portion of the supernatant was diluted with mass spectrometry-grade water to achieve a methanol content of 53%, followed by another centrifugation at 15,000 × g for 20 min at 4 °C, and the resulting supernatant was analyzed by LC-MS. Equal volumes of all samples were mixed to prepare a quality control (QC) sample, which was injected at the beginning, throughout, and at the end of the sample run to balance the chromatography-mass spectrometry system and monitor system stability during the entire analytical process. Procedural blank samples (53% methanol aqueous solution substituted for the biological sample) were processed and injected alongside study and pooled-QC samples; metabolite features whose peak area in the blank sample exceeded the corresponding peak area in the pooled QC samples were excluded from further analysis as likely background or carry-over artifacts.

*LC-MS/MS Detection:* The metabolite detection platform consisted of an Orbitrap Exploris™ 120 mass spectrometer (Thermo Fisher Scientific) coupled with a Vanquish UHPLC system. Data were acquired in both positive and negative ion modes. Chromatographic separation was performed using a Hypersil Gold C18 reverse-phase column (1.9 μm, 100 × 2.1 mm) at a column temperature of 40 °C and flow rate of 0.2 mL/min. Mobile phase A consisted of 0.1% formic acid in water, and mobile phase B consisted of methanol, with gradient elution. Data acquisition was performed in data-dependent acquisition (DDA) mode with positive/negative ion switching. Raw data were converted to mzXML format using ProteoWizard and processed using XCMS software for peak extraction and quantification. Differential metabolites were identified using the criteria of VIP > 1.0, FC > 1.2 or < 0.833, and *P* < 0.05, followed by KEGG pathway enrichment analysis of the differential metabolites. Benjamini-Hochberg false-discovery-rate (FDR) correction was additionally applied across all tested features to assess the robustness of this screening to multiple-testing correction.

### Microbiota-metabolite correlation analysis

Spearman correlation coefficients were calculated between differential bacterial genera and differential metabolites. Correlations were considered statistically significant at *P* < 0.05 and visualized as heatmaps.

### Statistical analysis

Continuous variables were presented as mean ± standard deviation (x ± s) and compared between groups using independent samples *t*-test or Mann-Whitney U test. Categorical variables were presented as frequencies (%) and compared using chi-square test or Fisher's exact test. All statistical analyses were performed using R software and IBM SPSS Statistics version 25.0. Statistical significance was defined as *P* < 0.05.

## Results

### Baseline characteristics of study subjects

One additional RCT participant was excluded from all the final analyses due to insufficient sample volume for sequencing/metabolomic testing. The RCT group comprised the remaining 60 patients (28 males, 32 females) with a mean age of 64.9 ± 7.4 years and mean BMI of 24.0 ± 2.6 kg/m^2^. The control group comprised 61 individuals (30 males, 31 females) with a mean age of 65.4 ± 7.5 years and mean BMI of 24.3 ± 2.8 kg/m^2^. There were no statistically significant differences between the two groups in baseline characteristics including age, sex, and BMI (all *P* > 0.05; Table 1).

**Table 1:** Comparison of baseline characteristics between RCT group and control group.

Variable	RCT group (*n* = 60)	Control group (*n* = 61)	*P* value
Age (years)	64.9 ± 7.4	65.4 ± 7.5	0.713
Sex (male/female)	28/32	30/31	0.782
BMI (kg/m^2^)	24.0 ± 2.6	24.3 ± 2.8	0.543

### Overall structural differences in gut microbiota

**Figure 1:**
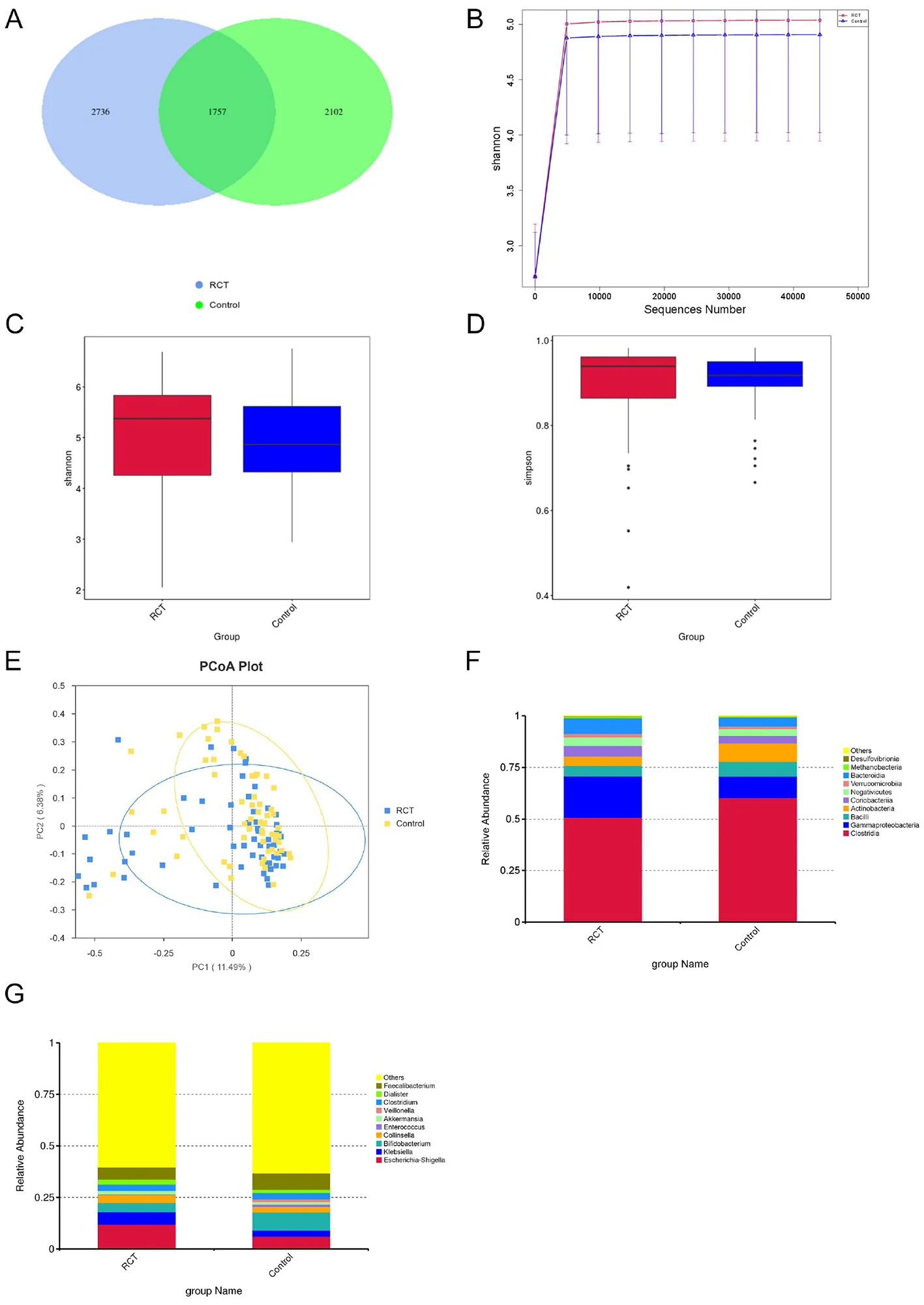
Characteristics of gut microbiota in patients with symptomatic degenerative rotator cuff tendinopathy. **(A)** Venn diagram showing shared and unique amplicon sequence variants (ASVs) between RCT and control groups. **(B)** Rarefaction curve assessing microbial sampling depth and coverage. **(C, D)** Alpha diversity analysis of microbiota in RCT and control groups using Shannon and Simpson indices. **(E)** Beta diversity shown by principal coordinate analysis (PCoA) based on weighted UniFrac distance, demonstrating differences in microbiota composition between the two groups. **(F, G)** Relative abundance of gut microbiota at phylum and genus levels in the two groups. RCT, symptomatic degenerative rotator cuff tendinopathy group; Control, control group.

All samples passed the sequencing quality threshold, generating 12,872,646 raw paired-end reads from 121 samples. After quality control filtering, chimera removal, and DADA2 denoising, a total of 6,630 ASVs were obtained. Each sample was rarefied to 44,069 reads for downstream diversity analysis. Venn diagram analysis of ASVs showed that the RCT group harbored 2,736 unique ASVs, the control group harbored 2,102 unique ASVs, and 1,757 ASVs were shared between the two groups, revealing substantial microbiota diversity specific to each group (Figure 1A). Rarefaction curve analysis demonstrated that all sample curves plateaued, confirming that the current sequencing depth adequately represented the species diversity in the samples (Figure 1B). Alpha diversity analysis revealed no statistically significant differences between the RCT and control groups in Shannon and Simpson diversity indices (all *P* > 0.05), indicating that the two groups had comparable overall species richness and evenness (Figures 1C, D). Principal coordinate analysis (PCoA) based on weighted UniFrac distance showed a separation trend between RCT and control group samples (ANOSIM, *R* = 0.026, *P* = 0.014; PERMANOVA/Adonis, *R*^2^ = 1.45%, *F* = 1.745, *P* = 0.007), indicating a statistically significant, albeit modest, difference in overall gut microbiota composition between patients with dRCT and controls—consistent with the substantial inter-individual variability typically observed in human gut microbiota studies (Figure 1E). At the phylum level, the major bacteria included *Bacillota, Pseudomonadota, Actinomycetota, Verrucomicrobiota, Bacteroidota, Methanobacteriota*, and *Thermodesulfovibrionota* (Figure 1F). At the genus level, the major bacteria included *Escherichia-Shigella, Klebsiella, Bifidobacterium, Collinsella, Enterococcus, Akkermansia, Veillonella, Dialister*, and *Faecalibacterium* (Figure 1G).

### LEfSe analysis of differential microbiota and microbial functional pathway prediction

**Figure 2:**
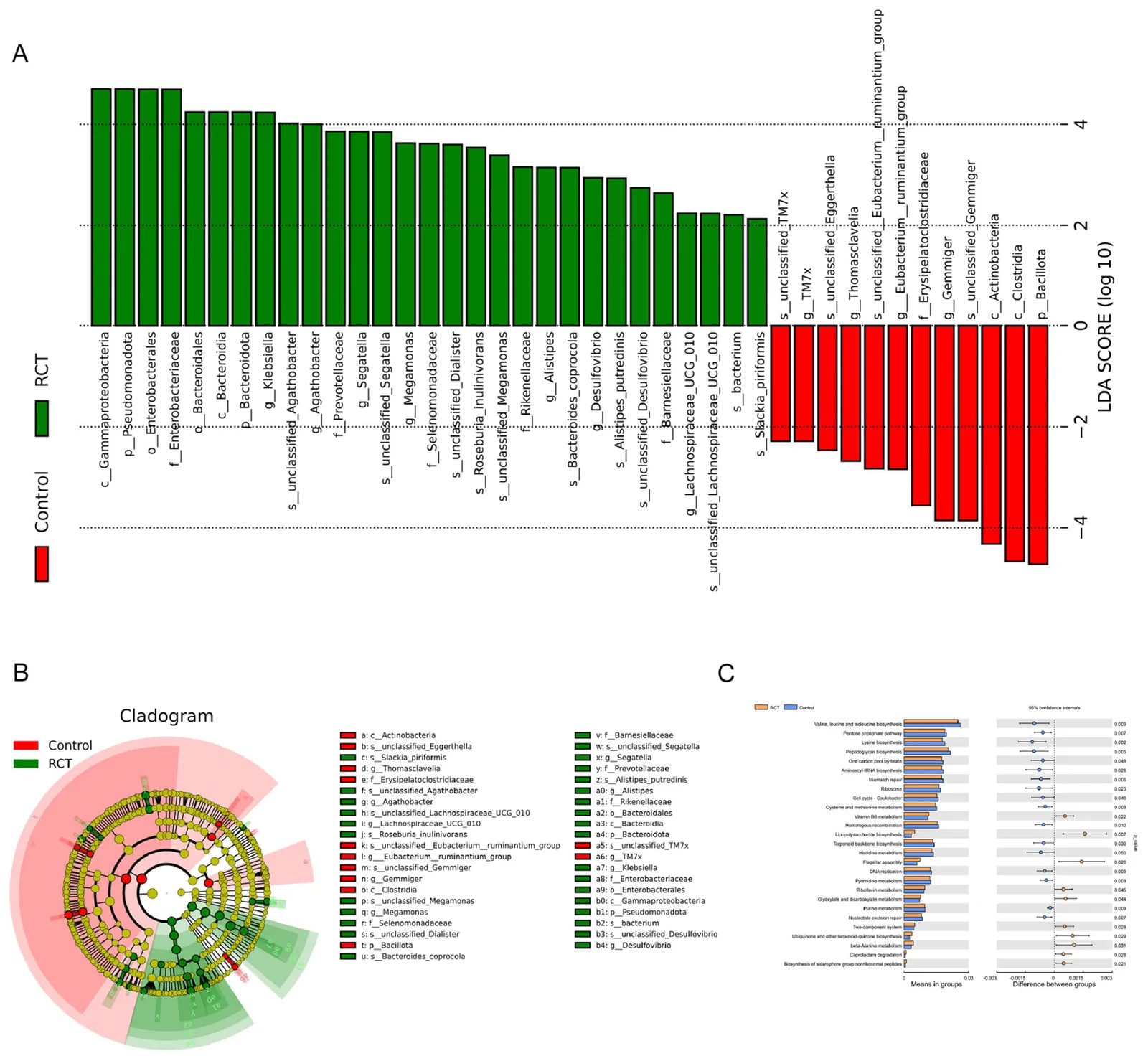
LEfSe effect size analysis and functional pathway prediction. **(A, B)** LEfSe analysis of gut microbiota composition between RCT and control groups (LDA > 2; *P* < 0.05) with corresponding phylogenetic trees. **(C)** PICRUSt2 prediction of microbial functional pathways. RCT, symptomatic degenerative rotator cuff tendinopathy group; Control, control group.

LEfSe analysis (LDA > 2) identified multiple taxonomic units with significant differences. At the phylum level, *Pseudomonadota* and *Bacteroidota* were significantly enriched in the RCT group; at the class level, *Bacteroidia* and *Gammaproteobacteria* were enriched; at the order level, *Bacteroidales* and *Enterobacterales* were enriched; at the family level, *Enterobacteriaceae, Prevotellaceae*, and *Rikenellaceae* were enriched; at the genus level, *Segatella, Megamonas, Klebsiella, Desulfovibrio, Agathobacter*, and *Alistipes* were significantly enriched (Figures 2A, B). In contrast, the control group was enriched in the phylum *Bacillota*, class *Clostridia* and *Actinobacteria*; at the genus level, *Gemmiger*, the *Eubacterium_ruminantium* group, and *Thomasclavelia* were enriched; at the family level, *Erysipelatoclostridiaceae* was enriched (Figures 2A, B).

Furthermore, based on 16S rRNA sequencing data, PICRUSt2 was employed to predict microbial functional pathways, identifying 28 KEGG metabolic pathways with significant differences between the two groups (all *P* < 0.05). The RCT group showed PICRUSt2-predicted enrichment in pro-inflammatory functional pathways including lipopolysaccharide biosynthesis, flagellar assembly, vitamin B6 metabolism, riboflavin metabolism, and glyoxylate and dicarboxylate metabolism. In contrast, the control group exhibited PICRUSt2-predicted greater functional activity in amino acid synthesis and tissue repair-related pathways, including valine, leucine, and isoleucine biosynthesis; lysine biosynthesis; peptidoglycan biosynthesis; pentose phosphate pathway; aminoacyl-tRNA biosynthesis; pyrimidine metabolism; purine metabolism; cysteine and methionine metabolism; and DNA mismatch repair (Figure 2C).

### Untargeted metabolomic analysis

*Overall metabolite differences and KEGG pathway enrichment analysis:*

**Figure 3:**
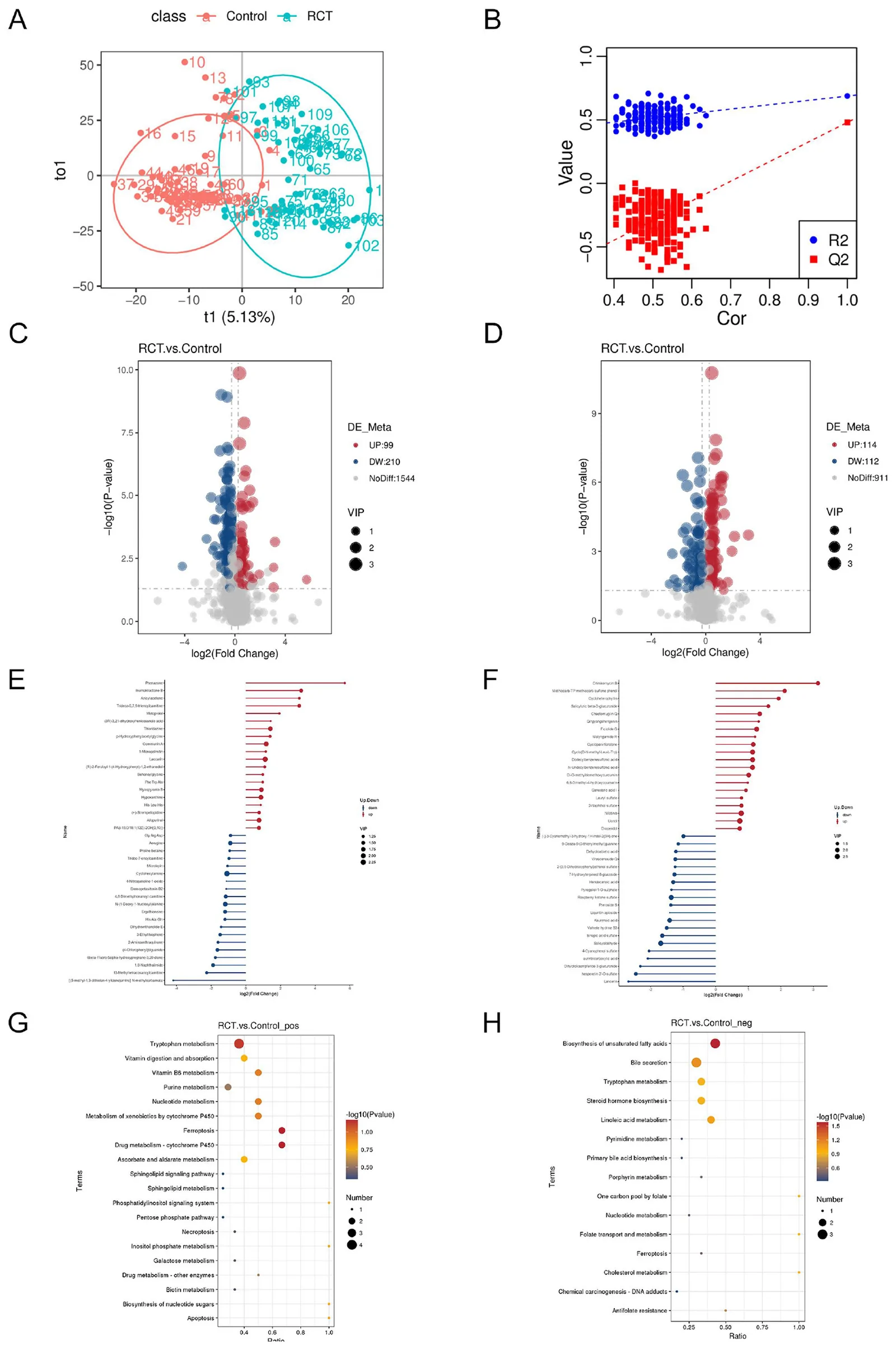
Differential metabolite analysis and pathway enrichment between RCT and control groups. **(A)** OPLS-DA score plots showing metabolic differences between the two groups. **(B)** Permutation plot of the OPLS-DA model validating model reliability and stability. **(C, D)** Volcano plots of significantly changed differential metabolites in positive ion mode **(left)** and negative ion mode **(right)**. **(E, F)** Bar plots of significantly changed differential metabolites in positive ion mode **(left)** and negative ion mode **(right)**. **(G, H)** Pathway enrichment analysis based on differential metabolic pathways between the two groups in positive ion mode **(left)** and negative ion mode **(right)**. OPLS-DA, orthogonal partial least squares discrimination analysis. RCT, symptomatic degenerative rotator cuff tendinopathy group; Control, control group.

Untargeted metabolomic analysis of plasma samples identified 1,853 metabolite features in positive ion mode, with 309 differential metabolites detected (99 upregulated and 210 downregulated in the RCT group); and 1,137 metabolite features in negative ion mode, with 226 differential metabolites detected (114 upregulated and 112 downregulated in the RCT group). Benjamini-Hochberg false-discovery-rate correction confirmed that 220/309 (71.2%) positive-ion-mode and 130/226 (57.5%) negative-ion-mode differential metabolites remained significant at FDR *q* < 0.05, indicating that the majority of these findings are robust to multiple-testing correction. OPLS-DA (orthogonal partial least squares discrimination analysis) score plots showed distinct clustering patterns between RCT and control groups (Figure 3A), and the validity and reliability of the OPLS-DA model were confirmed by permutation testing (Figure 3B). Volcano plots and bar plots displayed the differential metabolites showing significant changes in both positive and negative ion modes between the RCT and control groups (Figures 3C–F). KEGG enrichment analysis revealed that in positive ion mode, the most significantly altered pathways were concentrated in tryptophan metabolism, ferroptosis, drug metabolism-cytochrome P450, vitamin B6 metabolism, and vitamin digestion and absorption (Figure 3G). In negative ion mode, the most significant pathways included biosynthesis of unsaturated fatty acids, bile secretion, tryptophan metabolism, and steroid hormone biosynthesis (Figure 3H).

Among the differential metabolites, a coordinated panel of tryptophan-derived indole compounds was significantly downregulated in the RCT group, including indole-3-acetaldehyde (FC = 0.76, *P* = 6.7 × 10^−7^, FDR *q* = 1.4 × 10^−4^), indole-3-carboxaldehyde (FC = 0.82, *P* = 2.7 × 10^−4^, FDR *q* = 5.9 × 10^−3^), indolelactic acid (FC = 0.82, *P* = 2.1 × 10^−4^, FDR *q* = 5.1 × 10^−3^), indoleacrylic acid (FC = 0.81, *P* = 4.0 × 10^−4^, FDR *q* = 7.9 × 10^−3^), 3-methylindole (FC = 0.82, *P* = 5.4 × 10^−4^, FDR *q* = 9.9 × 10^−3^), indole (FC = 0.83, *P* = 8.8 × 10^−4^, FDR *q* = 1.3 × 10^−3^), and indoleacetic acid (FC = 0.76, *P* = 3.2 × 10^−3^, FDR *q* = 3.2 × 10^−2^). Vitamin E was also significantly downregulated (FC = 0.65, *P* = 1.3 × 10^−7^, FDR *q* = 3.9 × 10^−5^). Kynurenine-pathway intermediate xanthurenic acid was also significantly downregulated (FC = 0.79, *P* = 2.0 × 10^−3^, FDR *q* = 2.4 × 10^−2^). All remaining significant after FDR correction.

### Differential microbiota-metabolite correlation analysis

**Figure 4:**
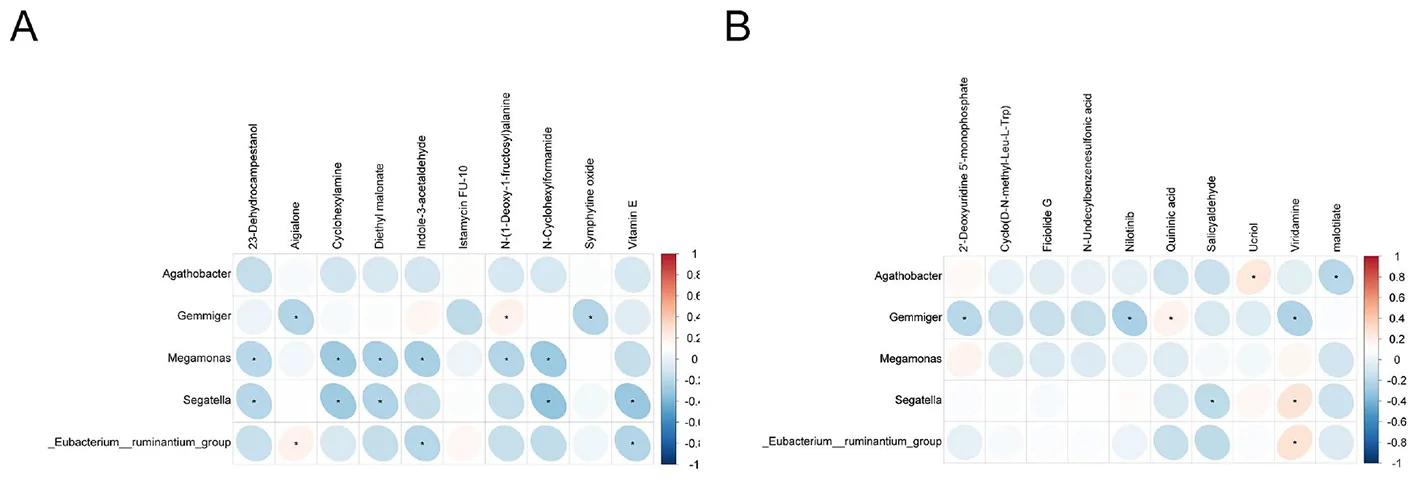
Spearman correlation analysis of differential microbiota and metabolites between RCT and control groups. **A** represents correlation analysis in positive ion mode; **B** represents correlation analysis in negative ion mode. Red indicates positive correlation; blue indicates negative correlation. *P < 0.05. RCT, symptomatic degenerative rotator cuff tendinopathy group; Control, control group.

Correlation analysis between differential bacterial genera and metabolites was performed to investigate their associations. In positive ion mode, the genera *Segatella* and *Megamonas* (enriched in the RCT group) showed negative correlations with indole-3-acetaldehyde and vitamin E. In negative ion mode, the genus *Gemmiger* (enriched in the control group) showed positive correlation with Quininic acid (Figure 4).

## Discussion

To our knowledge, this is the first multi-omics systematic investigation of the “gut-tendon axis.” By integrating 16S rRNA amplicon sequencing, untargeted metabolomic analysis, PICRUSt2 functional prediction, and microbiota-metabolite correlation analysis, we identified a characteristic pattern of gut microbiota dysbiosis in patients with symptomatic degenerative rotator cuff tendinopathy and propose a “gut-tendon axis”-related pathogenic hypothesis. We suggest that dysbiotic microbiota may be associated with tendon degeneration through multiple pathways, including enhanced production of gut-derived endotoxins, reduced tryptophan-to-indole conversion capacity, and ferroptosis-related oxidative stress.

Regarding the overall characteristics of gut microbiota, this study identified a typical “dysbiosis” pattern in patients with symptomatic degenerative rotator cuff tendinopathy: enrichment of pro-inflammatory bacteria accompanied by a divergent shift among butyrate-producing bacteria, with net depletion of the predominant taxa. This finding is highly consistent with recent studies in musculoskeletal system diseases, which demonstrate similar patterns of microbiota alterations in patients with osteoarthritis, osteoporosis, and spinal degeneration, suggesting that “pro-inflammatory-protective imbalance” may constitute a common microecological basis for chronic degeneration of the musculoskeletal system (Jiang et al., 2026; Plewa et al., 2026). A Mendelian randomization study by Yu et al. (2021) further supported a causal relationship between gut microbiota and osteoarthritis, providing important theoretical support for the current findings.

In the RCT group, *Klebsiella* was enriched. *Klebsiella* species are a hallmark marker of dysbiosis and serve as conditional pathobionts in the intestine. As gram-negative members of the Enterobacteriaceae family, their lipopolysaccharide (LPS) cell wall components can translocate across the damaged intestinal barrier (intestinal permeability defect or “leaky gut”) into blood circulation (Deng et al., 2026), and Klebsiella enrichment has been directly correlated with elevated pro-inflammatory cytokines (IL-6, TNF-α) and depletion of short-chain-fatty-acid-producing genera in a human case-control study (Ding et al., 2026). We hypothesize that a similar gut-derived pro-inflammatory burden may act on the local tendon microenvironment in dRCT patients. Multiple studies indicate that in the musculoskeletal system, inflammation promotes rotator cuff degeneration through the following mechanisms: (1) activation of macrophages to promote secretion of pro-inflammatory cytokines including TNF-α, IL-1β, and IL-6 (Liu et al., 2025); (2) regulation of matrix metalloproteinase (MMP-1, MMP-3, MMP-13, etc.) expression in tendon cells and synovial cells, accelerating collagen degradation and promoting rotator cuff degeneration (Connizzo and Grodzinsky, 2018; Jiang et al., 2023); (3) induction of oxidative stress and mitochondrial dysfunction in tendon cells, promoting apoptosis (Yu et al., 2026); (4) disruption of the normal remodeling balance at the tendon-bone interface (Jiang et al., 2024). *Segatella* was enriched in the RCT group. Research indicates that the potential role of *Segatella copri* in the pathogenesis of rheumatoid arthritis may be related to the stimulation of Th17 cell populations and the induction of Th17-associated cytokines, including IL-6 and IL-23 (Korzeniowska et al., 2024); Segatella copri itself has been directly shown to activate a pro-inflammatory macrophage profile via outer-membrane vesicles (Sepúlveda-Pontigo et al., 2025). Furthermore, *Megamonas*, enriched in the RCT group, has been demonstrated to be closely associated with systemic inflammation and metabolic syndrome (Sheng et al., 2022; Wu et al., 2024), suggesting that patients with degenerative rotator cuff tendinopathy may experience chronic low-grade systemic inflammation—a state that, in our interpretation, may represent an independent risk factor for tendon degeneration. Additionally, *Desulfovibrio* was enriched in the RCT group. *Desulfovibrio* belongs to sulfate-reducing bacteria and produces hydrogen sulfide (H_2_S), which has been confirmed to directly damage intestinal mucus layer integrity and exacerbate intestinal permeability (Kushkevych et al., 2020a,b). Previous studies have documented elevated *Desulfovibrio* abundance in patients with osteoarthritis and metabolic disorders (Ning et al., 2025; Sun et al., 2025), conditions that share epidemiological overlap with degenerative rotator cuff tendinopathy. Notably, butyrate-producing taxa did not show a uniform pattern: Agathobacter, itself a recognized butyrate producer, was enriched in the RCT group, whereas *Gemmiger* and the *Eubacterium_ruminantium* group, enriched in the control group, are both important butyrate-producing bacteria, and their enrichment suggests intact intestinal SCFA metabolism. Their significant reduction in the RCT group carries important pathophysiological significance. Butyrate, as the primary energy source for colonic epithelial cells, exerts anti-inflammatory and immunomodulatory effects by reinforcing intestinal epithelial barrier tight junctions, maintaining intestinal barrier integrity, inhibiting NF-κB-mediated inflammation, and promoting regulatory T cell differentiation (Furusawa et al., 2013; Peng et al., 2009). Although butyrate-producing bacteria exhibited a divergent trend, the decrease in Gemmiger and the Eubacterium_ruminantium group—the predominant butyrate-producing taxa—in the RCT group indicates a net reduction in SCFA production capacity, leading to increased intestinal permeability that may provide a pathway for endotoxins such as LPS to enter the bloodstream, forming a vicious cycle of “intestinal permeability–endotoxemia–systemic inflammation.”

The PICRUSt2 functional prediction results revealed that the gut microbiota in RCT patients was significantly active in lipopolysaccharide biosynthesis and flagellar assembly pathways, corresponding to the proposed “gut-derived endotoxemia” hypothesis. After translocating across the damaged intestinal barrier into systemic circulation, LPS binds to LPS-binding protein (LBP), inducing tendon cells to secrete pro-inflammatory cytokines including IL-1β, IL-6, and TNF-α, exacerbating damage and degeneration (Jeong et al., 2018). Research indicates that upregulation of flagellar assembly pathway is associated with enhanced bacterial motility and increased adhesion and invasion capability (Akahoshi and Bevins, 2022; Tran et al., 2019). In contrast, the control group's gut microbiota showed greater functional activity in branched-chain amino acid biosynthesis, lysine biosynthesis, and cysteine and methionine metabolism, which is consistent with the physiological function of healthy microbiota (represented by SCFA-producing bacteria) in supporting host protein synthesis and tissue repair. This finding has important implications for tissue repair: lysine is a critical amino acid constituent of type I collagen in tendons, and lysyl hydroxylase (LH) depends on lysine as substrate to accomplish collagen cross-linking and maintain tendon mechanical strength (Yamauchi and Sricholpech, 2012); branched-chain amino acids (BCAAs) promote protein synthesis through activation of the mTORC1 signaling pathway (Kaspy et al., 2024). Therefore, healthy microbiota sustains continuous metabolic substrate support for tendon collagen turnover and injury repair through maintenance of amino acid synthesis capacity, a function significantly impaired in dRCT patients.

Furthermore, in the untargeted metabolomic analysis, tryptophan metabolism pathway was significantly enriched in both positive and negative ion modes, representing the most reproducible core finding in this study. Tryptophan is metabolized primarily through three pathways: the kynurenine (Kyn) pathway, the serotonin pathway, and the indole pathway (Miao et al., 2025). The gut microbiota is the core determinant of tryptophan metabolic direction: healthy gut microbiota (*Lactobacillus, Bifidobacterium*, etc.) can convert tryptophan into indole and its derivatives (such as indole-3-acetaldehyde), which serve as aryl hydrocarbon receptor (AhR) ligands, activating the AhR-IL-22-intestinal barrier repair signaling axis and suppressing pro-inflammatory cytokine production. In contrast, when dysbiosis occurs, the Kyn pathway becomes excessively activated, producing large amounts of kynurenine and quinolinic acid, promoting systemic inflammation and oxidative stress (Cervenka et al., 2017; Roager and Licht, 2018). In this study, correlation analysis showed that the genera *Segatella* and *Megamonas*, enriched in the RCT group, were negatively correlated with indole-3-acetaldehyde, suggesting that enrichment of pro-inflammatory bacteria in dRCT patients may be associated with reduced conversion of tryptophan to indole-type metabolites, a pattern consistent with heightened systemic inflammation in tendon tissue. This mechanism has been confirmed in osteoarthritis and inflammatory bowel disease (Hua et al., 2025; Wei et al., 2023) and is here extended for the first time to the field of rotator cuff tendinopathy.

The significant enrichment of the ferroptosis pathway represents an innovative finding in our metabolomic analysis. Ferroptosis is a form of regulated cell death driven by iron-dependent lipid peroxidation, with core mechanisms involving inactivation of glutathione peroxidase 4 (GPX4) and peroxidation of polyunsaturated fatty acid (PUFA) phospholipids (Li et al., 2020). Recent studies have revealed that cells undergoing ischemia and oxidative stress are susceptible to ferroptosis, and GPX4 inhibition can accelerate cellular degeneration *in vitro*. The dysregulation of unsaturated fatty acid biosynthesis pathway provides critical lipid substrates for ferroptosis-related lipid peroxidation (Mou et al., 2019). In the RCT group, we simultaneously observed enrichment of the ferroptosis pathway and abnormal unsaturated fatty acid metabolism, suggesting that these two pathways may synergistically participate in tendon cell death and may represent an important molecular basis for irreversible tendon degeneration in dRCT—a direction warranting intensive future investigation.

The significant alterations in unsaturated fatty acid biosynthesis pathway carry multiple implications. Polyunsaturated fatty acids (PUFAs), particularly those of the omega-3 series, exert prominent anti-inflammatory and tendon-protective effects: suppressing the arachidonic acid cascade inflammatory pathway and promoting the production of resolvins and protectins (Bodur et al., 2025; Sandford et al., 2018). Reduction in PUFAs may lead to an imbalance between pro-inflammatory and anti-inflammatory lipid mediators, accelerating tendon degeneration. Additionally, changes in bile secretion pathway—represented by differential enrichment of taurocholic acid and bilirubin—suggest that gut microbiota may indirectly regulate the rate of tendon degeneration by affecting bile acid metabolism (FXR/TGR5 signaling axis). The bile acid profile is closely related to gut microbiota composition: the microbiota converts primary bile acids to secondary bile acids through bile salt hydrolase (BSH) and other enzymes, and dysbiosis can lead to reduced abundance of bacteria participating in bile acid biotransformation, resulting in decreased secondary bile acid levels (Larabi et al., 2023; Zhao et al., 2025). Furthermore, alterations in the steroid hormone biosynthesis pathway suggest that the gut microbiota may also indirectly regulate tendon degeneration rates by influencing the enterohepatic circulation of sex hormones (estrogen metabolism by bacterial β-glucuronidase), a mechanism already confirmed in osteoporosis (Guan et al., 2023; Lyu et al., 2023).

In this study, we performed correlation analyses in both positive and negative ion modes to determine associations between intestinal bacteria and metabolites. As noted earlier, in positive ion mode, the genera *Segatella* and *Megamonas*, enriched in the RCT group, showed negative correlations with indole-3-acetaldehyde, suggesting that enrichment of pro-inflammatory bacteria in dRCT patients may be associated with reduced conversion of tryptophan to indole-type metabolites, a pattern consistent with heightened systemic inflammation in tendon tissue. *Segatella* and *Megamonas*, enriched in the RCT group, showed significant negative correlations with vitamin E. Previous studies have confirmed that oxidative stress is one of the core pathological mechanisms in degenerative tendinopathy. The accumulation of reactive oxygen species (ROS) in tendon tissue activates matrix metalloproteinases (MMPs), inducing collagen fiber degradation and tendon cell apoptosis. Vitamin E, the principal lipid-soluble antioxidant, effectively scavenges reactive oxygen species (ROS), protecting tendon cells from oxidative stress damage, and maintains tendon collagen structure integrity through inhibition of lipid peroxidation (Millar et al., 2021; Xia et al., 2024). This suggests that dysbiosis-driven alterations in vitamin E bioavailability or metabolism in dRCT patients may increase the oxidative burden on the rotator cuff tendon. In negative ion mode, *Gemmiger*, enriched in the control group, showed a strong positive correlation with Quininic acid (FC = 0.68, *P* = 8.7 × 10^−8^, FDR *q* = 2.1 × 10^−5^, VIP = 2.47), among the most statistically robust associations identified in this analysis. Quininic acid is a quinoline-alkaloid-related compound whose endogenous role in human or gut-microbial metabolism has not, to our knowledge, been characterized; we are therefore cautious about proposing a specific mechanism and note this as a robust but mechanistically unexplained association warranting targeted validation in future studies. Overall, the correlation analysis results reveal potential associations between gut microbiota and metabolites that may significantly impact disease progression, though these remain speculative and require further mechanistic investigation to clarify these relationships.

The plausibility of a gut-tendon relationship is further supported by a recent murine study showing that postoperative treadmill exercise following rotator cuff repair altered gut microbiota composition and circulating metabolites, with Parabacteroides and its associated metabolites showing strong predictive value for tendon-bone healing outcomes (Wu et al., 2026). Although that study examined an exercise intervention during post-surgical recovery rather than baseline disease pathogenesis, it provides independent, mechanistically complementary evidence that gut microbial and metabolic status can influence rotator cuff tendon biology.

This study has numerous strengths, including the integration of multi-omics data analysis and the proposal of a “gut-tendon axis”-related pathogenic hypothesis. However, several limitations should be acknowledged: (1) The cross-sectional design cannot establish causality between gut microbiota and degenerative rotator cuff tendinopathy; future prospective cohort and mechanistic studies are needed; (2) The relatively limited sample size from a single research center may restrict the generalizability of findings, and statistical power requires further validation in larger populations; (3) 16S rRNA sequencing only reflects bacterial composition, and functional predictions (PICRUSt2) require validation through metagenomic or metatranscriptomic approaches and should be interpreted as hypothesis-generating rather than definitive evidence; (4) this study did not implement a standardized NSAID/analgesic washout protocol prior to sample collection, representing a potential unmeasured confounder given that NSAIDs and other analgesics are known to alter intestinal permeability and the mucosal microenvironment. This issue warrants further targeted investigation.

## Conclusion

This represents the first multi-omics systematic investigation of the “gut-tendon axis,” revealing that patients with symptomatic degenerative rotator cuff tendinopathy exhibit specific gut microbiota dysbiosis characterized by enrichment of pro-inflammatory bacteria and a divergent shift among SCFA/butyrate-producing bacteria, with net depletion of the predominant taxa. Our findings suggest that this dysbiosis may be associated with tendon degeneration through multiple pathways, including enhanced production of gut-derived lipopolysaccharides (endotoxins), an association with reduced tryptophan-to-indole conversion capacity, and ferroptosis-related oxidative stress. This study proposes a pathogenic hypothesis related to the “gut-tendon axis” and provides novel perspectives for the prevention and auxiliary treatment of degenerative rotator cuff tendinopathy. Targeted modulation of gut microbiota through SCFA supplementation, probiotics, and dietary interventions may represent new strategies for dRCT prevention and adjunctive therapy, awaiting further validation through future intervention studies.

## Data Availability

The 16S rRNA sequencing data presented in this study are deposited in the NCBI Sequence Read Archive (SRA) under BioProject accession number PRJNA1512149. The untargeted metabolomics data are deposited in the Open Archive for Miscellaneous Data (OMIX), National Genomics Data Center, under accession number OMIX019696.
